# Supplementary material for: Human Hematopoietic Stem Cell Engrafted IL-15 Transgenic NSG Mice Support Robust NK Cell Responses and Sustained HIV-1 Infection
Source: Viruses. 2023 Jan 27;15(2):365. doi: 10.3390/v15020365 (PMC9960100; doi:10.3390/v15020365)
Supplement: Supplementary file 1 [file viruses-15-00365-s001.zip › viruses-2076817-supplementary.pdf]

Supplemental Table S1: Flow Cytometry Antibodies

Target	Fluorophore	Clone	Catalog Number	Company
CD45	BV605	HI30	304042	Biolegend
CD56	BV570	HCD56	318330	Biolegend
CD16	PE-Cy5	3G8	302010	Biolegend
CD3	APC/Fire 750	UCHT1	300470	Biolegend
CD4	BV650	OKT4	317436	Biolegend
CD8a	BV785	RPA-T8	301046	Biolegend
CD19	BV750	HIB19	302262	Biolegend
CD11b	PE/Dazzle 594	ICRF44	301348	Biolegend
CD11c	PE/CY7	3.9	46-0116-42	Thermo
CD14	BV510	MΦP9	563079	BD
CD66b	PE	G10F5	305106	BD
mCD45*	AF700	30-F11	103128	Biolegend
CD34	BV480	8G12	746688	BD
FoxP3	AF532	2H7	58-0209-41	Invitrogen
Tbet	BV711	4B10	644820	Biolegend
EOMES	PerCP-eFluor 710	WD1928	46-4877-42	Invitrogen
GATA3	BV421	16E10A23	16E10A23	Biolegend
RORYT	AF647	Q21-559	563620	BD
TNF-a	BV750	MAb11	566359	BD
IFN-g	AF700	4S.B3	502520	Biolegend
Perforin	PE	B-D48	353304	Biolegend
Granzyme B	PacBlue	GB11	515408	Biolegend
CXCR6	PE/Dazzle 594	K041E5	356016	Biolegend
CD62L	PerCP-Cy5.5	DREG-56	304824	Biolegend
CD57	BB515	NK-1	565285	BD
CD69	BV510	FN50	310936	Biolegend
CD49e	BV650	IIA1	740586	BD
CD107a/Lamp1	BV786	H4A3	563869	BD
NKp46	BV650	9E2	331927	Biolegend
NKp30	BUV805	P30-15	749127	BD
KIR3DL1	PerCP/Cy5.5	DX9	312718	Biolegend
KIR2DL1	AF488	143211	FAB1844G	R&D
KIR2DL3	AF700	180701	FAB2014N	R&D
KIR2DS4	AF647	179315	564375	BD

* Murine specific antibodies, all others are specific to human antigen

Supplemental Figure S1

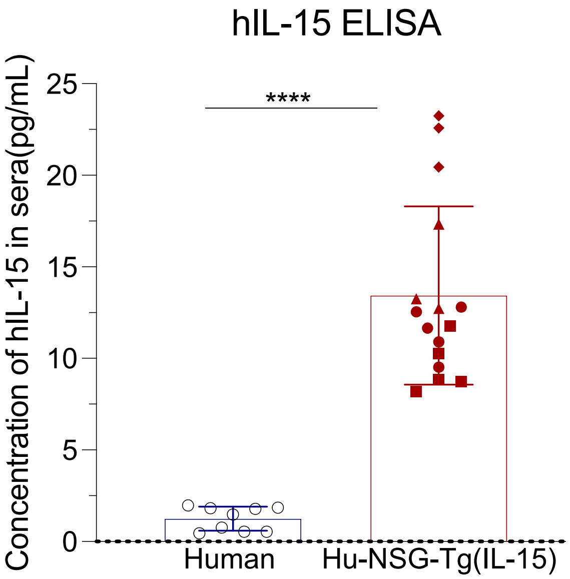

Supplemental Figure S1: Concentration of human IL-15 in sera of humanized NSG-Tg(IL-15) (n=16, 4 unrelated donors) and human donors (n=9). Statistical significance was calculated using an unpaired t-test.

Supplemental Figure S2

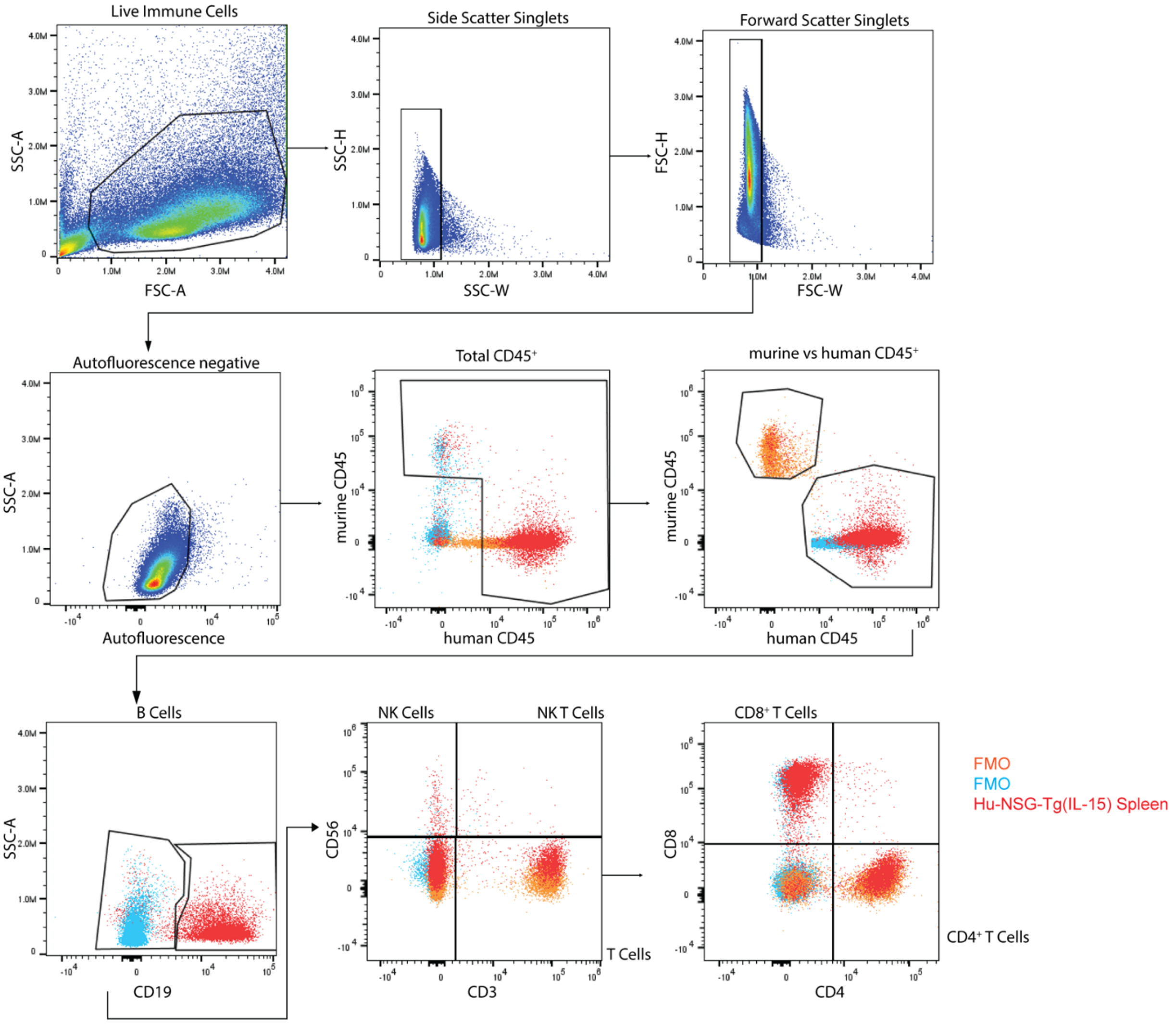

Supplemental Figure S2. Gating Strategy: Fluorescence minus one (FMO) control staining for each fluorophore in each respective panel is displayed in orange and teal. Representative Hu-NSG-Tg(IL-15) spleen staining in red. Markers of different cell types as detailed in the figure.

Supplemental Figure S3

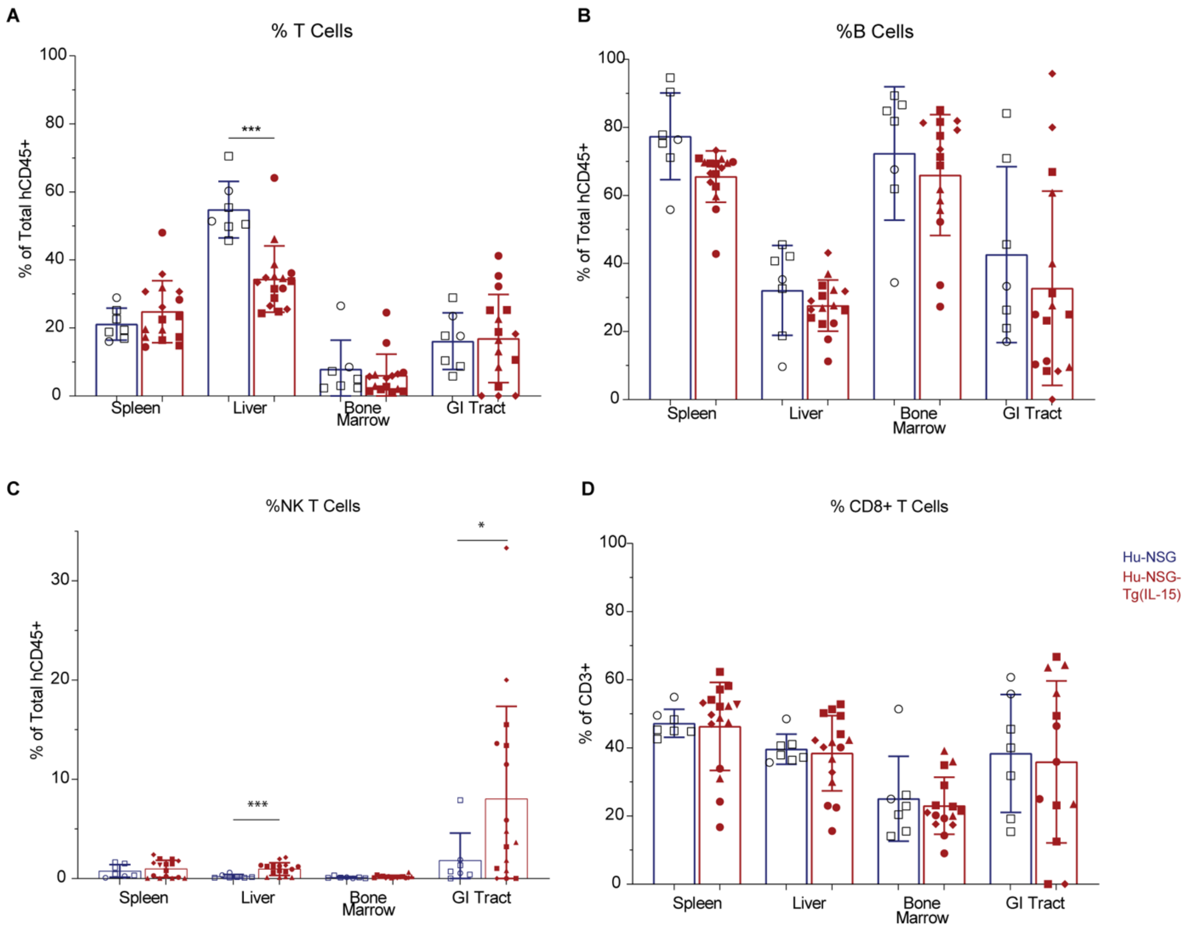

Supplemental Figure S3: Hu-NSG-Tg(IL-15) and Hu-NSG mice engraftment of T cells, B cells and NK T cells across multiple organs. A) Percentages of human T cells ($CD3^+CD56^-CD19^-$) in PBMC, spleen, liver, bone marrow, and gastrointestinal tract. Hu-NSG (n=7, 2 unrelated donors) and Hu-NSG-Tg (IL-15) (n=16, 4 unrelated donors). B) Percentage of human B cells ($CD3^-CD56^-CD19^+$) in PBMC, spleen, liver, bone marrow, and gastrointestinal tract. Hu-NSG (n=7, 2 unrelated donors) and Hu-NSG-Tg(IL-15) (n=16, 4 unrelated donors). C) Percentage of human NK T cells ($CD3^+CD56^+CD19^-$) in PBMC, spleen, liver, bone marrow, and gastrointestinal tract. Hu-NSG (n=7, 2 unrelated donors) and Hu-NSG-Tg(IL-15) (n=16, 4 unrelated donors). D) Percentage of human $CD8^+$ T cells ($CD3^+CD8^+CD4^-CD56^-CD19^-$) in PBMC, spleen, liver, bone marrow, and gastrointestinal tract. Hu-NSG (n=7, 2 unrelated donors) and Hu-NSG-Tg (IL-15) (n=16, 4 unrelated donors). Statistical significance was calculated using unpaired t-test for 2 groups *$P < 0.05$, and ***$P < 0.005$.

Supplemental Figure S4

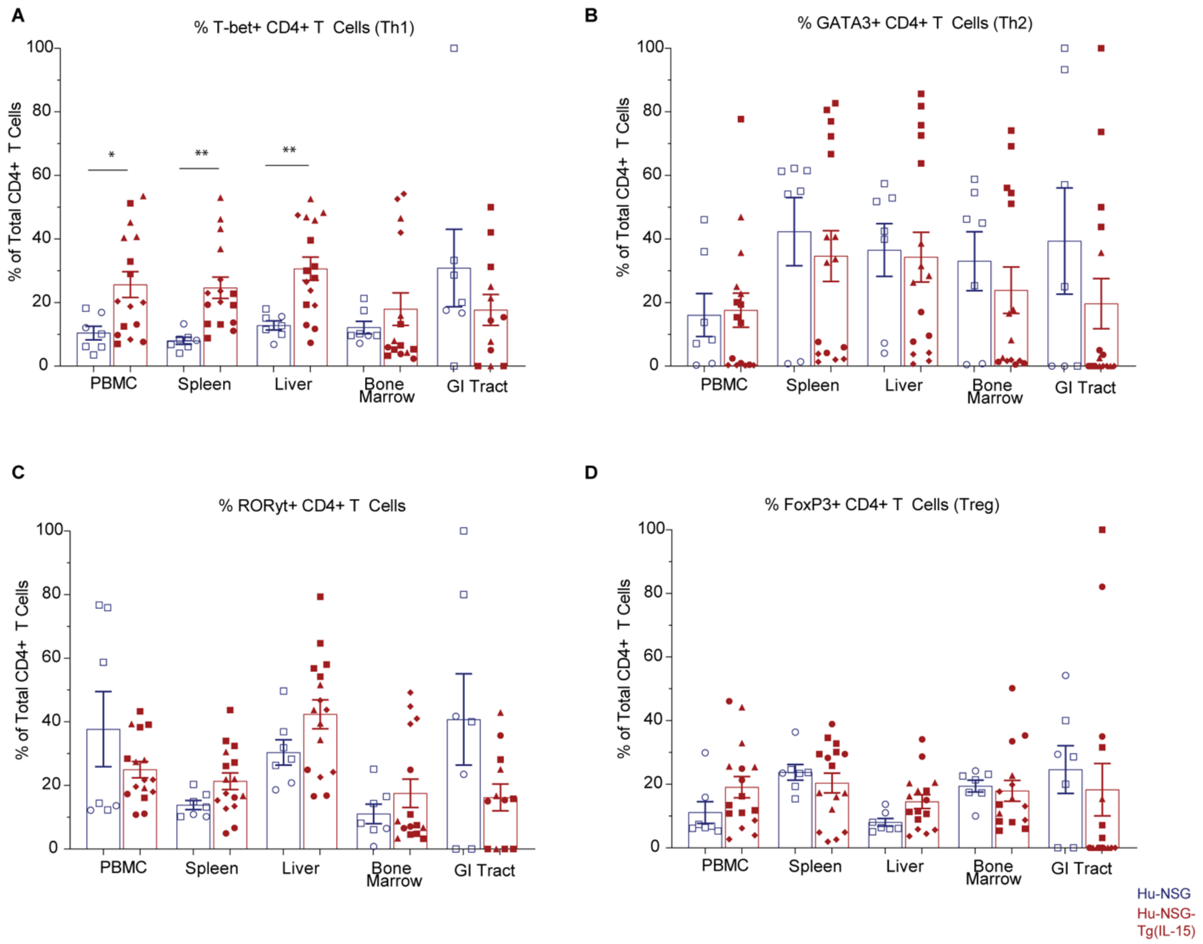

Supplemental Figure S4: Hu-NSG-Tg(IL-15) and Hu-NSG mice engraftment of $CD4^+$ T cell subsets across multiple organs. A) Percentage of $T\text{-}bet^+$ T cells ($CD3^+CD4^+Tbet^+CD8^-CD56^-CD19^-$) in PBMC, spleen, liver, bone marrow, and gastrointestinal tract. Hu-NSG (n=7, 2 unrelated donors) and Hu-NSG-Tg(IL-15) (n=16, 4 unrelated donors). B) Percentage of $GATA3^+$ T cells ($CD3^+CD4^+GATA3^+CD8^-CD56^-CD19^-$) in PBMC, spleen, liver, bone marrow, and gastrointestinal tract. Hu-NSG (n=7, 2 unrelated donors) and Hu-NSG (IL-15Tg) (n=16, 4 unrelated donors). C) Percentage of $ROR\gamma T^+$ T cells ($CD3^+CD4^+ROR\gamma T^+CD8^-CD56^-CD19^-$) in PBMC, spleen, liver, bone marrow, and gastrointestinal tract. Hu-NSG (n=7, 2 unrelated donors) and Hu-NSG-Tg(IL-15) (n=16, 4 unrelated donors). D) Percentage of $FoxP3^+$ T cells ($CD3^+CD4^+FoxP3^+CD8^-CD56^-CD19^-$) in PBMC, spleen, liver, bone marrow, and gastrointestinal tract. Hu-NSG (n=7, 2 unrelated donors) and Hu-NSG-Tg-(IL-15) (n=16, 4 unrelated donors). Statistical significance was calculated using unpaired t-test for 2 groups *$P < 0.05$, *$P<0.01$, and ***$P < 0.005$.

Supplemental Figure S5

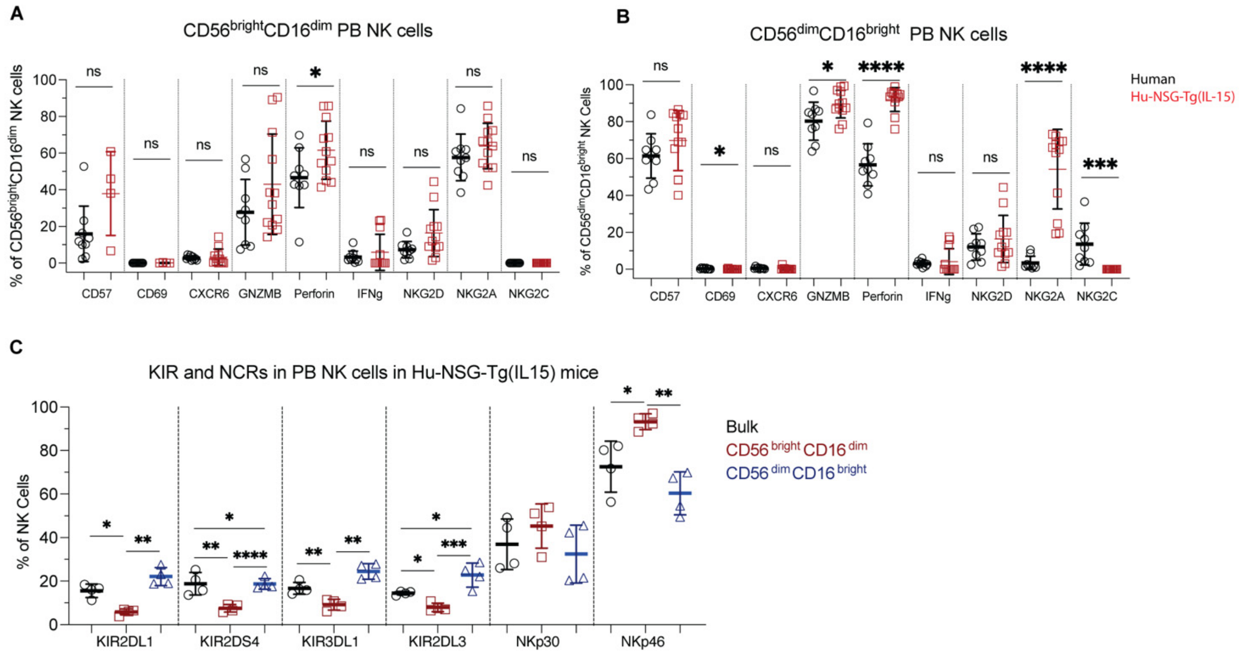

Supplemental Figure S5: Phenotypic characterization of CD56^{bright}CD16^{dim} and CD56^{dim}CD16^{bright} peripheral blood NK cells subsets in Hu-NSG-Tg(IL-15). A) Percentage of CD57, CD69, CXCR6, Granzyme B (GNZMB), Perforin, IFNg, NKG2D, NKG2A or NKG2C positive cells in the CD56^{bright}CD16^{dim} subset of peripheral blood NK cells in human donors (n=9) and Hu-NSG-Tg(IL-15) mice (1-3 unrelated donors, n=4-10 mice per donor, 3-6 months post CD34^{+} HSC transplant). B) Percentage of CD57, CD69, CXCR6, Granzyme B (GNZMB), Perforin, IFNg ,NKG2D, NKG2A or NKG2C positive cells in the CD56^{dim}CD16^{bright} subset of peripheral blood NK cells in in human donors (n=9) and Hu-NSG-Tg(IL-15) mice (1-3 unrelated donors, n=4-10 mice per donor, 3-6 months post CD34^{+} HSC transplant). C) Percentage of KIR and NCR receptors on total PB NK cells (Bulk), CD56^{bright}CD16^{dim} PB NK cells and CD56^{dim}CD16^{bright} PB NK cells from Hu-NSG-Tg(IL-15) mice (1 donor, n=4 per group) 4 months post-transplant with CD34^{+} HSCs. Statistical significance was calculated using unpaired t-test for 2 groups and one-way ordinary ANOVA with multiple comparions for 3 groups. *P < 0.05, *P<0.01, ***P < 0.005 and ****P<0.001.

Supplemental Figure S6

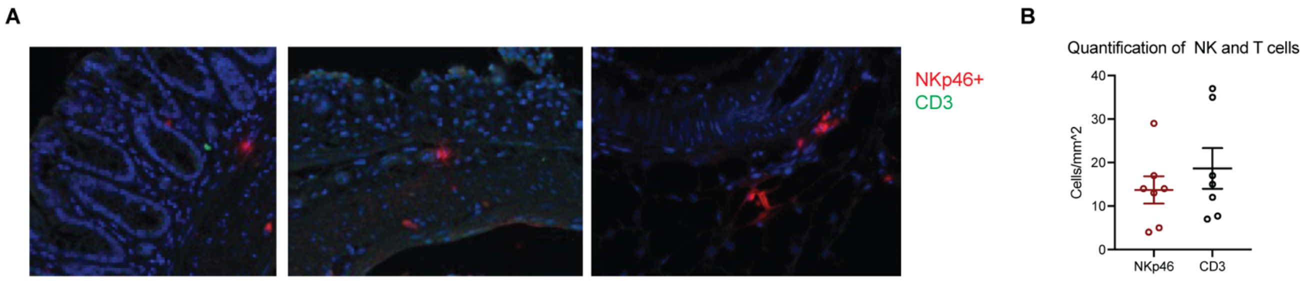

Supplemental Figure S6: NK Cell and T cell Immunohistochemistry of the GI tract of healthy Hu-NSG-Tg(IL-15) mice. A) Immunohistochemistry of FFPE NK cells (NKp46⁺) shown in red and T cells (CD3⁺) shown in green from GI tract of Hu-NSG(IL-15Tg) mice (n=7) 6 months post CD34⁺ HSC transplant. B) Quantification of IHC stained NKp46⁺ cells and CD3+ cells of Hu-NSG(IL-15Tg) mice 6 months post CD34⁺ HSC transplant.

Supplemental Figure S7

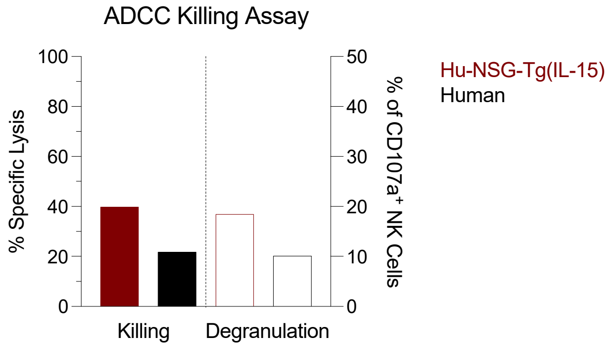

Supplemental Figure S7: Antibody-dependent cellular cytotoxicity killing assay. Calcein release assay with splenocytes from Hu-NSG-Tg(IL-15) or human PBMCs co-cultured with Raji cells at 10:1 effector to target ratio for 6 hours in the presence of Anti-CD20 antibody (left axis). Cells from 2 mice were pooled prior to incubation with target cells. Background cell death during co-culture in the absence of anti-CD20 was deducted. Hu-NSG-Tg(IL-15) mouse splenocytes or human PBMCs were stained with anti-CD107a antibody after 6 hours co-cultured with Raji cells (right axis). CD107a⁺ expressing NK cells (hCD45⁺mCD45⁻CD3⁻CD56⁺CD107a⁺) as a percentage of total NK cells is presented.

Supplemental Figure S8

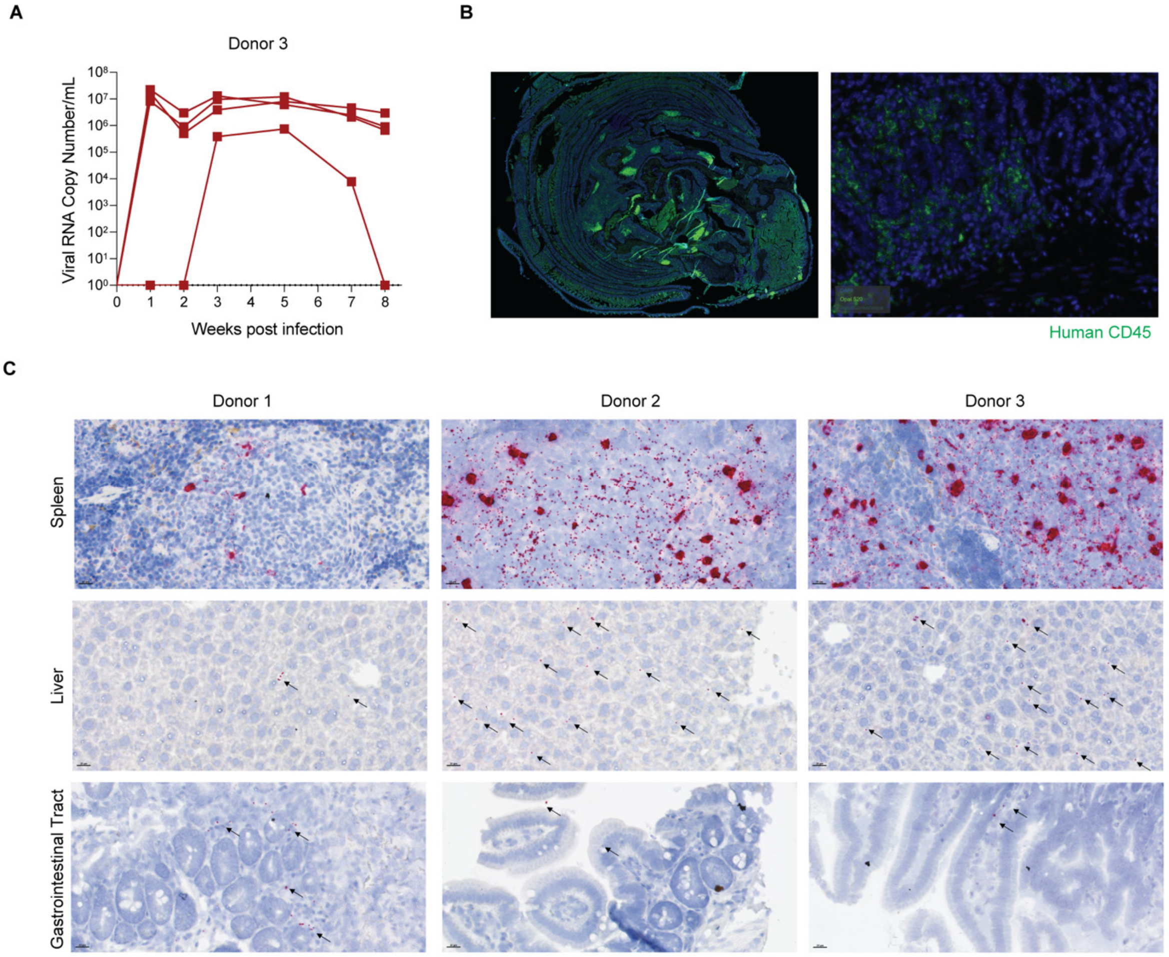

Supplemental Figure S8 : Hu-NSG-Tg(IL-15) mice display human immune cells in the GI tract and HIV-1 infection in the blood, spleen, liver and GI tract across multiple donor cohorts. A) RT-qPCR of viral RNA extracted from sera of HIV-1 infected (Q23.17 10^5 IU) Hu-NSG-Tg(IL-15) mice from donor 3 (n=4 mice) 6 months post CD34$^+$ stem cell transplant. B) IHC of human immune cells in GI tract harvested from Hu-NSG-Tg(IL-15) mouse 8 weeks post-infection. C) Representative images from RNAscope performed on FFPE blocks of spleen, liver, and GI tract harvested 8 weeks post infection with HIV-1 from Hu-NSG-Tg(IL-15) mice for 3 donors. Red punctate dots indicate positive binding of HIV-1 probe to HIV-1 RNA. Arrows indicate positive RNAscope staining.
